# Relationship between the Increased Haemostatic Properties of Blood Platelets and Oxidative Stress Level in Multiple Sclerosis Patients with the Secondary Progressive Stage

**DOI:** 10.1155/2015/240918

**Published:** 2015-04-29

**Authors:** Agnieszka Morel, Michał Bijak, Elżbieta Miller, Joanna Rywaniak, Sergiusz Miller, Joanna Saluk

**Affiliations:** ^1^Department of General Biochemistry, Faculty of Biology and Environmental Protection, University of Lodz, Pomorska 141/143, 90-236 Lodz, Poland; ^2^Department of Physical Medicine, Medical University of Lodz, Plac Hallera 1, 90-647 Lodz, Poland; ^3^Neurorehabilitation Ward, III General Hospital in Lodz, Milionowa 14, 93-113 Lodz, Poland; ^4^Department of Orthodontics, Medical University in Lodz, Pomorska 251, 92-213 Lodz, Poland; ^5^Department of Toxicology, Faculty of Pharmacy with Division of Medical Analytics, Wroclaw Medical University, Borowska 211, 50-556 Wroclaw, Poland

## Abstract

Multiple sclerosis (MS) is the autoimmune disease of the central nervous system with complex pathogenesis, different clinical courses and recurrent neurological relapses and/or progression. Despite various scientific papers that focused on early stage of MS, our study targets selective group of late stage secondary progressive MS patients. The presented work is concerned with the reactivity of blood platelets in primary hemostasis in SP MS patients. 50 SP MS patients and 50 healthy volunteers (never diagnosed with MS or other chronic diseases) were examined to evaluate the biological activity of blood platelets (adhesion, aggregation), especially their response to the most important physiological agonists (thrombin, ADP, and collagen) and the effect of oxidative stress on platelet activity. We found that the blood platelets from SP MS patients were significantly more sensitive to all used agonists in comparison with control group. Moreover, the platelet hemostatic function was advanced in patients suffering from SP MS and positively correlated with increased production of O_2_^−∙^ in these cells, as well as with Expanded Disability Status Scale. We postulate that the increased oxidative stress in blood platelets in SP MS may be primarily responsible for the altered haemostatic properties of blood platelets.

## 1. Introduction

Multiple sclerosis (MS) is a chronic autoimmune, inflammatory, and demyelinating disease involving demyelination of nerve sheath and disintegration of axons of the central nervous system (CNS), leading to disturbances of neurotransmission processes and, consequently, to occurrence of the neurological symptoms. On the clinical and pathological grounds, MS is a heterogeneous disease, and therefore different biological pathways may be active in different MS patients [1].

Clinically there are four main subtypes of MS: relapsing-remitting (RR MS), primary progressive (PP MS), secondary progressive (SP MS), and also progressive relapsing (PR MS) [2].

The most prevalent form of MS is RR MS, where disease fluctuates between periods of inflammation/demyelination and remission. Finally, after several years of the disease duration, RR MS in approximately 70% of cases turns into a secondary progressive disease in which patients suffer irreversible disability progression [3]. The progressive phase of multiple sclerosis depends on neuronal degeneration and cortical atrophy [4]. Accumulated data indicates that oxidative stress (OS) plays a critical role in this process [5, 6].

Other mechanisms responsible for the disease development in patients with SP MS have not been definitely recognized yet, although OS resulting in mitochondrial injury might also participate in the induction of demyelination and neurodegeneration in progressive stages of MS. OS, in both the relapsing-remitting and the progressive stages of MS, seems to be primarily powered by inflammation and oxidative burst in microglia; however, its effects might get amplified in patients with SP MS by age-dependent iron accumulation in the brain as well as by mitochondrial gene deletions, elicited by the chronic inflammatory process [7].

The MS duration is one of the main risk factors of stroke and deep vein thrombosis [8]. It has been shown that the coagulation cascade, leading to the generation of large amounts of thrombin responsible also for platelet activation, may play a key role in the development of inflammation in MS [9]. The recent data also indicates that blood platelets could be a potential therapy target in MS, since they are implicated in the development of neuroinflammatory process associated with this disorder. Various compounds stored in platelet *α*-granules can affect the permeability of BBB and are crucial for the infiltration of T-lymphocytes which are responsible for the dissemination of new inflammatory lesions in the nervous system in RR MS [10]. Many physiological agonists activate platelets and, consequently, cause their adhesion, secretion of compounds stored in their granules, microparticle formation, and receptor expression which finally induces platelet aggregation. The excessively activated blood platelets by endogenous agonists contribute to the disturbances in various diseases [11]. The coagulation cascade itself plays a major role in the development of an inflammatory response in MS [8]. Thrombin is the major coagulation factor of blood clotting cascade responsible for conversion of fibrinogen to fibrin [12], as well as the most potent activator of platelets, responsible for prothrombotic platelet function [12]. Proteomic studies of laser-captured microdissected lesions reported in the transcriptional profiling in MS [13] illuminated a key role for the thrombin cascade in the development of inflammation in MS. However, the triggering mechanism for platelet activation process and the role of platelets in hemostasis in a further development of the disease in the secondary progressive stage are still not clear.

Our previous studies show that OS might be a very important phenomenon in MS [14, 15]. As it has been known, OS is involved in many chronic diseases, such as neurodegenerative and cardiovascular disorders. The overproduction of reactive oxygen specials (ROS), observed as a major driving factor of demyelination and neurodegeneration in progressive MS [3], has important pathophysiological implications and may modulate physiological response of blood platelets (which are involved in hemostasis). ROS are implicated in regulation of platelet function and may be produced as second messengers, in the receptor-mediated signaling pathways during platelet activation.

In the presented work, we focused on one course of MS to make our work more precise in this heterogenic disorder. Therefore, our study assessed the adhesion of thrombin-stimulated and nonstimulated human blood platelets to collagen or fibrinogen. In our studies, we determined the reactivity of blood platelets evaluated by their ability to aggregate, upon stimulation with different strong agonists: ADP and collagen.

Another goal of our study was to show the link between ROS generation and platelet hyperactivation in secondary progressive stage of MS. In addition to the tests for the biological activity of blood platelets, we examined the level of OS in platelets by measuring the concentration of superoxide anion radicals generated in these cells. We correlated the level of O_2_^−∙^ formed in blood platelets with hemostatic activity of platelets expressed as their adhesion and aggregation. In blood platelets mainly O_2_^−∙^ is generated. It can scavenge endothelial-derived NO in a fast reaction generating peroxynitrite (ONOO^−^), a significant pathogenic factor that is responsible for neuron damage to neuron in MS.

## 2. Materials and Methods

### 2.1. Demographic and Clinical Characteristics

The blood samples were collected from 50 patients (male *n* = 22; female *n* = 28), suffering from secondary progressive (SP) course of MS. The patients were observed for one year before the blood collection. When initial relapsing-remitting course is followed by progression, with or without occasional relapses, minor remissions, and plateaux, the SPMS can be recognized. McDonald's criteria were used to diagnose the MS. The clinical parameters in patients with MS are mean age of 48.2 ± 15.2 years, disability status scale (EDSS) of 5.5 ± 1.8 and mean disease duration of 14.3 ± 8.3 years, and modified Rankin scale of 2–4. The blood samples were delivered from Neurological Rehabilitation Division III General Hospital in Lodz, Poland.

The control blood samples were obtained from fifty healthy volunteers (male *n* = 19; female *n* = 31), not taking any medications, who have never been diagnosed with MS or other chronic diseases and without any neurological or hormonal illness and any chronic inflammatory disease. The control groups and patients with MS (Table 1) were matched by the age and sex.

These two populations (control and MS) were statistically compared, which confirmed the homology between these groups in age, BMI, and gender.

The protocol and all procedures were done according to Helsinki Declaration and were approved by Ethics Committee of the Medical University of Lodz, Poland, RNN/260/08/KB.

### 2.2. Isolation of Human Blood Platelets

The blood samples were collected into CPDA-1 (citrate phosphate dextrose adenine-1), taken from a peripheral vein between 8 and 9 am. The blood platelets were isolated by differential centrifugation of blood as described by Wachowicz and Kustroń (1992) [16] and counted by the photometric method according to Walkowiak et al. (1989) [17]. The platelets were washed and resuspended in modified Tyrode's (Ca^2+^/Mg^2+^) free buffer (127 mM NaCl, 2.7 mM KCl, 0.5 mM NaH_2_PO_4_, 12 mM NaHCO_3_, 5 mM HEPES, 5.6 mM glucose, and pH 7.4).

### 2.3. Reagents

ADP was obtained from Chrono-Log Corporation (Havertown, PA), divided into small 500 *μ*mol/L stock aliquots and stored at 2–8°C until use (for adhesion and aggregation). Collagen type I, bovine serum albumin (BSA), and bicinchoninic acid (BCA) solution were delivered from Sigma (St. Louis, MO, USA). Thrombin was purchased from BioMed (Lublin). Fibrinogen was prepared from citrated human plasma, by the combination of cold and ethanol precipitations technique by Doolittles' method [18].

### 2.4. Platelet Adhesion

The adhesion of blood platelets to fibrinogen or collagen type I was determined according to Tuszyński and Murphy methods [12]. The platelet adhesion measurement was based on ELISA method. The first step was to coat each well of Nunc microplatelet (MaxiSorp) by the protein coat, fibrinogen or collagen. The application volume of the fibrinogen or collagen was 100 *μ*L per well for a 96-well plate at the final concentration of 0.1 U/mL. The coat microplatelet was incubated for 16 h at 4°C. After incubation, to remove the unbound proteins, the microplatelet was washed three times with 200 *μ*L of PBS. Then, 200 *μ*L of 1% bovine albumin was added onto the coated wells. The microplatelet was incubated for 2 h at 37°C. The excess of bovine albumin was poured off and the microplate was washed three times with 200 *μ*L of PBS. To each well, 100 *μ*L of platelet suspension (3 × 10^8^ platelets/mL) was added. In order to activate the blood platelets, 50 *μ*L of thrombin at the final concentration of 0,6 U/mL was added to the platelet suspension. The microplatelet was incubated for 2 h at 37°C. The nonadherent cells were removed by washing with 200 *μ*L of PBS. To determine the total protein concentration, each well was incubated for 1 h at 37°C with 200 *μ*L of Sigma BCA working solutions and after incubation the microplatelet was spectrophotometrically measured at 562 nm with a platelet reader.

### 2.5. Platelet Aggregation

The whole blood was centrifuged for 10 min at 250 ×g at room temperature to get platelet-rich-plasma (PRP). The platelet aggregation was measured in platelet-rich-plasma (PRP) by turbidimetric method using the optical Chrono-Log aggregometer (Chrono-Log, Havertown, PA). After preincubation of PRP (3 × 10^8^ platelets/mL) at 37°C for 5 min, the agonist solutions were added: ADP (10 *μ*M) or collagen (2 *μ*g/mL). The aggregation was measured with stirring by the duration of 10 minutes. The results are presented as a percentage of the aggregation. The maximal aggregation (100%) was defined as the maximum change in light transmission, observed in PPP (platelet-poor-plasma).

### 2.6. O_2_^−∙^ Generation

The generation of superoxide anion radicals (O_2_^−∙^) in SP MS and in the control platelets was measured by the cytochrome c reduction, as described earlier [19]. For that purpose, one mL of cytochrome c (160 *μ*M) prepared in Ca^2+^/Mg^2+^ free Tyrode's buffer was added to an equal volume of platelet suspensions in the same buffer. After incubation, the platelets were sedimented by centrifugation at 2000 ×g for 5 min and the supernatants were added to cuvettes. The reduction of cytochrome c was measured spectrophotometrically at 550 nm. To calculate the molar concentration of O_2_^−∙^, an extinction coefficient for cytochrome c of 18700 M^−1^ cm^−1^ was used [20].

### 2.7. Statistical Analysis

The results were statistically elaborated. The values were expressed as means ± SD. The Shapiro-Wilk test was used for checking the normality of the distribution of examined data. Student's *t*-test was used for the normal distribution or Mann-Whitney *U* test when the distribution was nonnormal.

The analysis of correlation parameters for the superoxide anion generation in blood platelets, the platelets adhesion to fibrinogen/collagen, and the aggregation were estimated by Spearman's rank correlation. For all correlations, Spearman's rank correlation coefficient was estimated and plot regression was performed. Similarly, we analyzed the dependence of platelet aggregation and the Expanded Disability Status Scale, as well as the relationship between EDSS and Beck Depression Inventory.

The statistical analysis was performed using StatsDirect Statistical software version 2.7.2. *p* < 0.05 was considered as statistically significant.

## 3. Results

**Figure 1:**
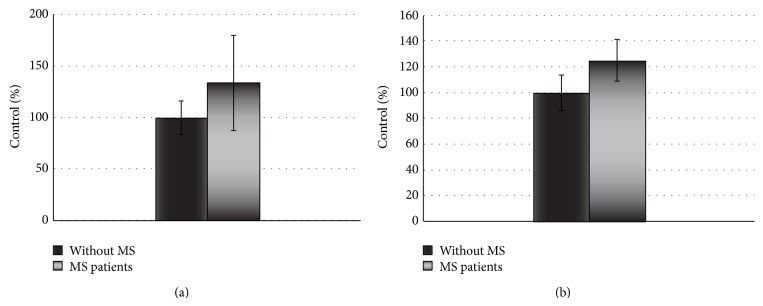
The adhesion of resting platelets to fibrinogen (2 mg/mL) (a) or collagen (0.1 U/mL) (b). The data are presented as means ± SD (*n* = 50; ^∗^ *p* < 0.0001 SP MS platelets versus control (without MS) by Mann-Whitney *U* test).

**Figure 2:**
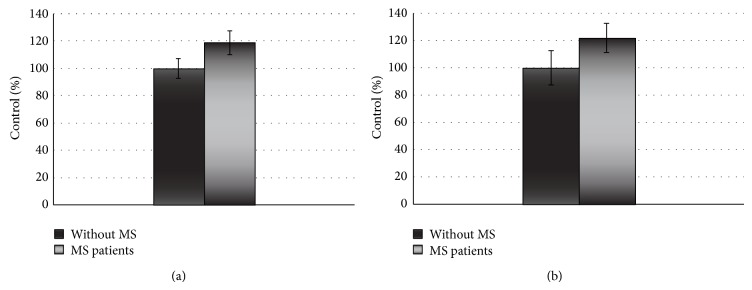
The adhesion of thrombin-activated platelets to fibrinogen (2 mg/mL) (a) or collagen (0.1 U/mL) (b). The data are presented as means ± SD (*n* = 50; ^∗^ *p* < 0.0001 SP MS versus control (without MS) by Mann-Whitney *U* test).

**Figure 3:**
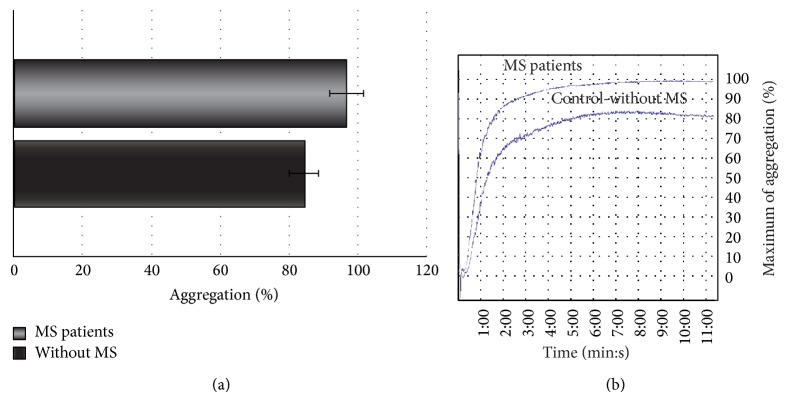
The blood platelet aggregation induced by ADP in platelet-rich-plasma (PRP). (a) The data are presented as means ± SD (*n* = 50; ^∗^ *p* < 0.0001 SP MS platelets versus control (without MS) by Mann-Whitney *U* test). (b) The typical curve of platelet aggregation after stimulation of platelets by ADP (Chrono-Log aggregometer, Havertown, PA).

**Figure 4:**
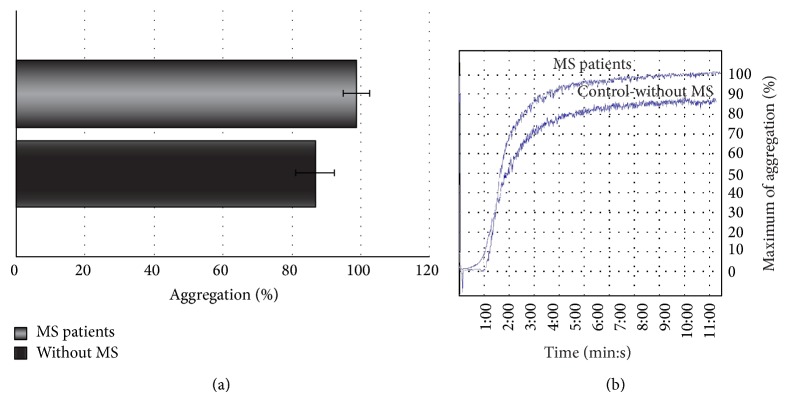
The blood platelet aggregation induced by collagen in platelet-rich-plasma (PRP). (a) The data are presented as means ± SD (*n* = 50; ^∗^ *p* < 0.0001 SP MS platelets versus control (without MS) by Mann-Whitney *U* test). (b) The typical curve of platelet aggregation after stimulation of platelets by ADP (Chrono-Log aggregometer, Havertown, PA).

Our results indicate that platelets from SP MS patients show statistically significant increase of adhesion (Figures 1 and 2) and aggregation (Figures 3 and 4).

The adhesion of blood platelets obtained from both healthy controls and SP MS patients is shown in Figures 1 and 2. Our results demonstrate that the adhesion of blood platelets to fibrinogen (Figures 1(a) and 2(a)) and to collagen type I (Figures 1(b) and 2(b)) was markedly higher in platelets from patients with SP MS than in healthy subjects. The platelets obtained from SP MS, both unstimulated (resting) (Figures 1(a) and 1(b)) and stimulated with 0.6 U/mL thrombin (Figures 2(a) and 2(b)), showed the distinctly enhanced adhesion (*p* < 0.0001). The adhesion level of SP MS was about 20–35% higher in comparison with the control platelets. The adhesion of control platelets from healthy subjects was expressed as 100%. We also observed the statistically significant enhancement of the aggregation of platelets obtained from SP MS group compared to healthy control (Figures 3 and 4). The level of platelet aggregation upon ADP stimulation increased from 83% for healthy controls to 98% for SP MS patients (growth of 18% of control, when the value of the control was taken as 100%) (Figure 3(a)). The aggregation induced by collagen was enhanced from 87.5% for healthy persons to 99% for SP MS patients (the 13% growth of control) (Figure 4(a)).

**Figure 5:**
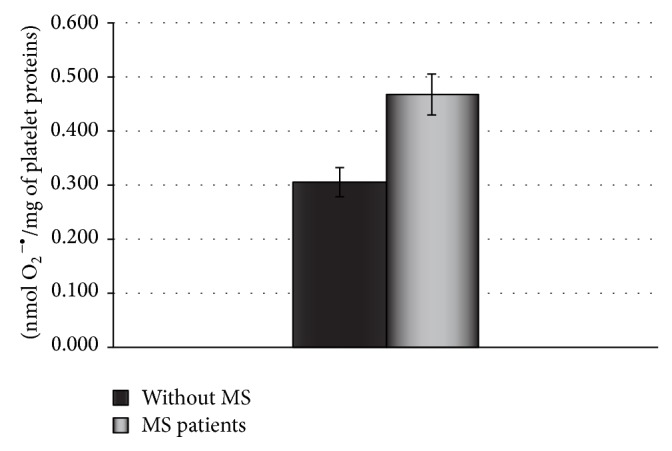
The generation of superoxide anion radicals in blood platelets from SP MS patients. The results are done in triplicate and are expressed as means ± SD (*n* = 50, ^∗^ *p* < 0.001 versus control (without MS) by Student's *t*-test).

The other set of experiments involved the measurement of the level of O_2_^−∙^ generation in blood platelets. The concentration level of O_2_^−∙^ was statistically significantly elevated in blood platelets obtained from SP MS patients compared to healthy volunteers (*p* < 0.001). The level of O_2_^−∙^ reached a mean value of 0.305 nmol/mg of platelet proteins (SD = 0.03) in platelets of SP MS patients while for healthy controls the level reached a mean value of 0.469 nmol/mg of platelet proteins (SD = 0.04), respectively (Figure 5).

**Figure 6:**
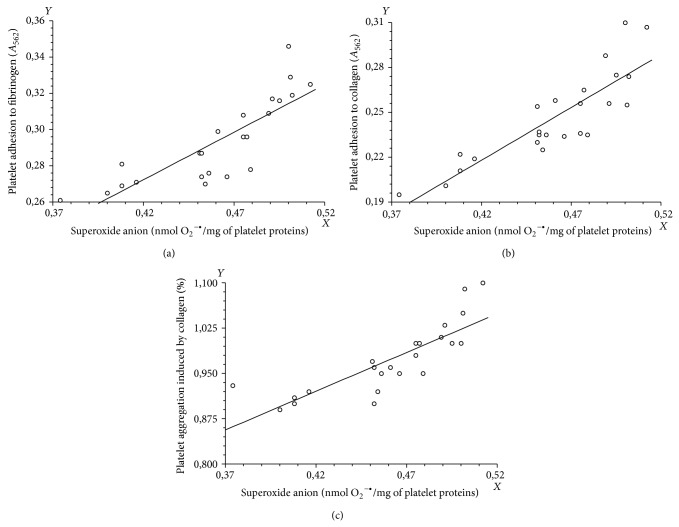
The correlation analysis between the superoxide anion level in blood platelets and the platelet adhesion to fibrinogen (a) or collagen (b) or the platelet aggregation induced by collagen (c) (Spearman's rank correlation).

Importantly, we found statistically significant positive correlation between the increase of thrombin-stimulated blood platelets adhesion and degree of O_2_^−∙^ generation in blood platelets in SP MS patients (Figures 6(a) and 6(b) and Table 2), as well as between collagen-induced platelet aggregation and O_2_^−∙^ generation in blood platelets in SP MS patients (Figure 6(c) and Table 2). In all cases, we observed statistically significant (*p* < 0.0001) positive correlation between the superoxide anion generation and the platelet adhesion or aggregation. Spearman's rank correlation coefficients were, respectively, 0.88001 for platelet adhesion to fibrinogen, 0.860597 for platelet adhesion to collagen, and 0.881833 for the platelet aggregation (Table 2).

**Figure 7:**
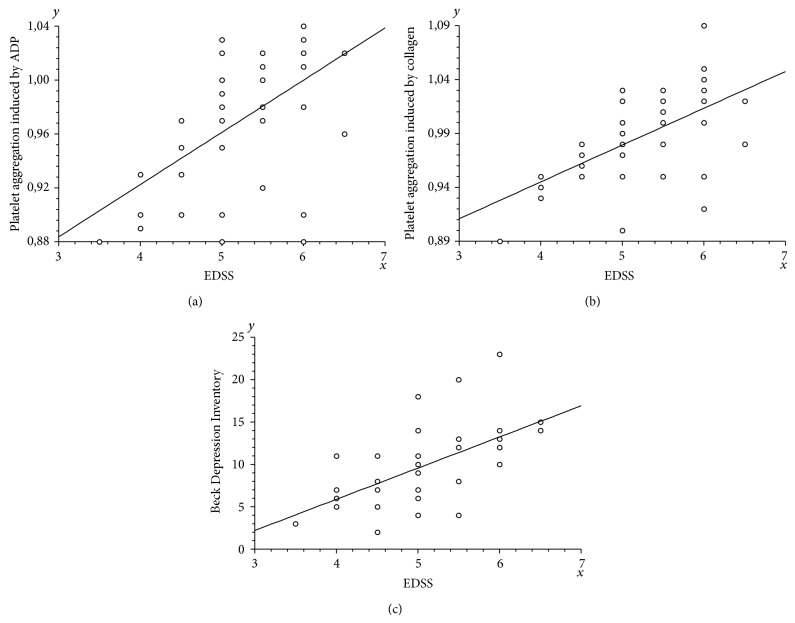
The correlation analysis between the Expanded Disability Status Scale and the platelet aggregation induced by ADP (a) or collagen (b) or the Beck Depression Inventory (c) (Spearman's rank correlation).

We also demonstrated the relationship between Expanded Disability Status Scale (EDSS) and blood platelet aggregation level. Statistically significant (*p* < 0.0001) positive correlation was observed between EDSS and platelet aggregation induced by both ADP and collagen (Spearman's rank correlation coefficients were, resp., 0.568162 and 0.610421) (Figures 7(a) and 7(b) and Table 3).

Additionally, we checked the dependence of EDSS and other clinical parameters. The analysis showed the statistically significant (*p* < 0.0001) positive correlation (Rho = 0,674675) between EDSS and Beck Depression Inventory (Figure 7(c)).

In this study, we did not find statistically significant correlation between plasma concentrations of investigated compounds and age, BMI, and mean disease duration in SP MS patients.

## 4. Discussion

The coagulation cascade plays the critical role in the development of an inflammatory response in MS. Langer et al. [21] proved that platelets are trapped in chronic active demyelinating MS lesion. They also observed that, after inhibiting the main platelets receptors, GP IIb/IIIa, the paralysis and experimental autoimmune encephalomyelitis were, respectively, ameliorated and reduced. Such findings are a great opportunity to consider that the glycoprotein IIb/IIIa blockers, like Abciximab, may play a crucial role in MS and other demyelinating diseases.

The proteomic studies pointed out a great role of thrombin cascade in the development of inflammation in MS [9, 22].

The blood platelets are the main elements of the cellular hemostasis and also play an important role in the coagulation cascade. These multifunctional cells are activated by different endogenous, physiological agonists, including ADP, collagen, or thrombin, due to the vast number of receptors present on the surface of platelets. Upon vessel wall injury circulating platelets are immediately immobilized by interactions with vWF bounding to collagen and the glycoprotein GPIb-V-IX complex [23] which initiates adhesion of flowing platelets to the subendothelial extracellular matrix (ECM).

In the present study, we focused on the primary hemostasis in patients with SP MS. Our studies were designed to demonstrate the changes in platelet hemostatic function of patients suffering from secondary progressive multiple sclerosis in comparison with healthy control group.

In the 1950s, there were a few early studies on the role of blood platelets in the central nervous system demyelination, which showed the increased adhesion of platelets in the MS [23]. Since that time, next papers were published confirming the platelet abnormalities in MS patients [24]. The studies of Sheremata et al. [25] demonstrated that blood platelets are significantly activated in MS patients [25]. To investigate the basis of these observations, the authors have applied the flow cytometric analysis to measure the platelet-derived microparticles (PMP) and platelet microaggregates formation, as well as an expression of platelet activation marker CD62P (P-selectin) [25]. Their findings indicate the elevated platelet activation in clinically stable relapsing-remitting MS patients and confirm the conclusion presented in the earlier literature that platelets are chronically activated in RR MS.

The significant role of activated platelets in the development of inflammatory response is directly related to their adhesion to inflamed changed endothelial cells or proteins presented in the subendothelial layer of blood vessel wall and to tendency of blood platelets to form aggregates with leukocytes [26, 27]. The platelets are able to directly activate both leukocytes and dendritic cells. There have been reports that suggested the hypothesis that the interaction of platelets with leukocytes and endothelial cells is directly responsible for the disruption of the BBB and considered as the key initial step of the disease process in some neurological inflammatory diseases, including MS, leading to infiltration of lymphocytes and further to the formation of inflammatory lesions in the brain [28].

All of cited reports emphasize the importance of platelet activation in MS. However, as highlighted by the authors of these studies [25], the role of platelets in pathogenesis of MS remains unknown, and the further studies are required to validate the significance of the existing study and to determine the role of platelet activation in neuropathogenesis of MS. In addition, there are very few data describing the physiology of platelets in the secondary progressive phase of MS. Among the existing reports, the differences in platelet activation parameters between RR MS and SP MS exist. Higher level of sP-selectin and other markers of platelet activation [29] as well as an increase of PAF activity [30] were observed in RR MS compared to secondary progressive MS. Due to the fact that each clinical phase of MS should be considered as a distinct disease entity [31], we focused our attention on the alterations of platelets in SP MS characterized by irreversible disability progression [3].

Our results demonstrate that the activation of platelets from SP MS patients is significantly elevated compared to the activation of platelets from healthy subjects. The level of platelet aggregation induced by physiological agonists (ADP, collagen) and adhesion to the two key adhesion proteins (collagen and fibrinogen) in patients with SP MS is greater than in the control group. We clearly indicate that SP MS platelets are more sensitive to agonists and their response is significantly stronger than platelets obtained from the healthy subjects. Our observation of chronically increased platelet activation is consistent with other studies.

The oxidative stress has been implicated in the pathophysiology of MS, but its relation to disease progression as well as to the functioning of hemostasis is uncertain. The studies of Bö et al. [32] implicate the free radical nitric oxide role in the pathogenesis of demyelinating MS lesions because of the markedly elevated human inducible nitric oxide synthase in tissue sections from MS [32]. In the fast reaction between ^∙^NO and O_2_^−∙^, short-lived species capable of inducing oxidative/nitrative changes in a wide variety of biomolecules are produced. Among them, the highest reactivity exhibits peroxynitrite (PN, ONOO^−^) [33]. PN is an important pathogenetic factor in MS, which causes the increased activity of inflammatory processes. ONOO^−^ generated in the circulatory system is a major redox imbalances factor involved in the disturbance of hemostasis. It is responsible for damage of plasma components and cells [1]. Many pathogenic processes, including blood platelet hyperactivity, may be initiated by injury action of free radical species [34]. During platelet activation mainly O_2_^−∙^ and other ROS are produced [35]; therefore, blood platelets link the processes of hemostasis, thrombosis, and inflammation [36].

It is possible that the platelet intercellular mechanisms of activation where ROS take place may be modulated during progression of disease status in MS. However, we are not sure if the activation of blood platelets in SP MS is simply a consequence of general inflammation and overproduction of reactive oxygen and nitrogen species. The epidemiological data highlight the high risk of cardiovascular disease in the course of MS [37]. Thus, it is possible that the increased platelet activation may be a component of procoagulant state in this disease. We observed a positive correlation between the level of O_2_^−∙^ (measured by the method of cytochrome c reduction) in blood platelets and two steps of their activation, for example, amplified adhesion and elevated aggregation (Figure 6 and Table 2).

The superoxide radicals may interact with iron (accumulated in the brain in SP MS) and form reactive hydroxyl radicals that attack polyunsaturated fatty acids, leading to lipid peroxidation and demyelination. Moreover, based on the hypothesis that mitochondrial injury, induced by ROS/RNS, is a major driving factor of demyelination and neurodegeneration in progressive MS [3], we might postulate that excessive platelet reactivity in SP MS is the result of increased OS in blood platelets. In progressive MS, in chronic active and inactive lesions, the number of mitochondria is increased and the enzymatic activity of the respiratory chain is altered [38, 39].

## 5. Conclusion

We measured the platelet aggregation and adhesion as the markers of hemostatic activity of blood platelets. Our findings demonstrate the changes of platelet hemostatic function in patients suffering from SP MS in comparison with healthy subjects, who have never been diagnosed with MS or other chronic diseases. In our study, we have shown that platelet activity in patients with SP MS is significantly higher than in healthy controls. Our results demonstrate that platelet reactivity and aggregation induced by ADP or collagen in blood platelets from SP MS patients are greater than aggregation of platelets obtained from healthy volunteers. Furthermore, blood platelet aggregation is positively correlated with the degree of disability of patients. The level of platelet aggregation induced by major physiological agonists (ADP, collagen) has reached, respectively, 90% and 98% in patients with SP MS. We also observed a significant increase in platelet adhesion to collagen or fibrinogen (30% in comparison with control group). These findings suggest that platelets from SP MS patients are significantly more sensitive to the key physiological agonists, which may be an important cause of hemostatic disorders occurring in SP MS.

Moreover, our results show the positive correlation between the level of O_2_^−∙^ generation and changes of platelet hemostatic responses in SP MS patients.

Considering the data presented in this study, we suggest that blood platelets actively participate in oxidative stress existing in SP MS. The production of O_2_^−∙^ in these cells, one of the main substrates for the formation of peroxynitrite, can adjust the changes in platelet hemostasis in progressive stage of MS. Further investigations are continuing.

Understanding platelet pathophysiology provides real new therapeutic targets in MS. It seems that blood platelets are important determinants in the MS pathogenesis, but the use of antiplatelet agents (inhibiting platelet activation) requires the inclusion of antioxidants, especially in progressive stages of disease.

The role of platelet in MS especially during different subtypes of MS required further studies.

## Figures and Tables

**Table 1:** The characteristics of study subjects and control groups.

	Healthy controls	SPMS
	(*n* = 50)	(*n* = 50)
Mean age [years]	45.7 ± 10.2	48.2 ± 15.2
Gender [*n* (frequency])		
Male	19	22
Female	31	28
EDSS	—	5.5 ± 1.8
Mean disease duration [years]	—	14.3 ± 8.3

EDSS: Expanded Disability Status Scale; SPMS: secondary progressive multiple sclerosis.

**Table 2:** The correlation parameters values obtained for blood platelet hemostatic functions and degree of O_2_^−•^ generation in samples from SP MS patients. The rank correlation coefficients and probability of correlation are presented.

	Blood platelet haemostatic functions					
	The adhesion of thrombin-stimulated platelets to fibrinogen		The adhesion of thrombin-stimulated platelets to collagen		Platelet aggregation induced by ADP	
	Rho	*p*	Rho	*p*	Rho	*p*
O_2_^−•^	0.88001	*p* < 0.0001	0.860597	*p* < 0.0001	0.8818	*p* < 0.0001

Rho: rank correlation coefficient.

**Table 3:** The correlation parameters values obtained for Expanded Disability Status Scale and blood platelet aggregation level. The rank correlation coefficients and probability of correlation are presented.

	Blood platelet haemostatic functions			
	Platelet aggregation induced by ADP		Platelet aggregation induced by collagen	
	Rho	*p*	Rho	*p*
EDDS	0.568162	*p* < 0.0001	0.610421	*p* < 0.0001

EDSS: Expanded Disability Status Scale; Rho: rank correlation coefficient.
